# NAD ^+^ Metabolic Reprogramming Drives CD8 ^+^ T Cells Senescence and Exacerbates Ulcerative Colitis

**DOI:** 10.1111/acel.70706

**Published:** 2026-09-09

**Authors:** Maolin Ye, Qi Zhou, Mingjia Kong, Suhan Zhao, Lingxi Lin, Lirong Chen, Jianghong Yu, Feifei Luo, Jie Liu, Jun Zhang

**Affiliations:** ^1^ Department of Digestive Diseases Huashan Hospital, Fudan University Shanghai China; ^2^ Shanghai Institute of Materia Medica Chinese Academy of Sciences Shanghai China; ^3^ National Clinical Research Center for Aging and Medicine Huashan Hospital, Fudan University Shanghai China

**Keywords:** immunosenescence, inflammatory bowel disease, NAD^+^, senolytic therapy

## Abstract

While immunosenescence is increasingly implicated in chronic inflammatory disorders, its precise pathogenic contribution to ulcerative colitis (UC) remains elusive. Here, we identify senescent CD8^+^ T cells as a distinct pathogenic population that exacerbates colitis, demonstrating that systemic senolytic treatment significantly attenuates disease severity. Mechanistically, nicotinamide adenine dinucleotide (NAD^+^) metabolic dysregulation triggers mitochondrial dysfunction and cytosolic mitochondrial DNA leakage, promoting CD8^+^ T cell senescence through the activation of the cGAS‐STING signaling pathway. Spatial transcriptomic mapping reveals that senescent CD8^+^ T cells are enriched within mucosal niches experiencing NAD^+^ metabolic dysregulation. Crucially, this senescent‐metabolic signature correlates with severe disease phenotypes and predicts non‐response to biologic therapies in UC patients. Collectively, our findings uncover a critical NAD^+^‐cGAS‐STING axis driving T cell senescence, establishing the clearance of senescent immune cells as a promising therapeutic strategy for UC.

## Graphical abstract

Nicotinamide adenine dinucleotide (NAD^+^) depletion induces mitochondrial dysfunction and cytosolic mtDNA leakage in CD8^+^ T cells, activating the cGAS‐STING pathway and promoting a senescence‐associated secretory phenotype (SASP). The resulting pro‐inflammatory CD8^+^ T cells release IFN‐γ and TNF‐α, disrupt the intestinal epithelial barrier, and exacerbate ulcerative colitis.

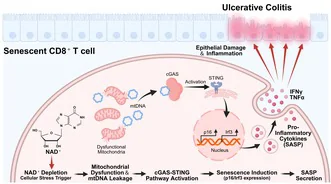

## 1 Introduction

Immunosenescence, traditionally viewed as an age‐dependent deterioration of immune function, is increasingly recognized as a contributor in numerous chronic inflammatory disorders (Hu et al. 2025; Yousefzadeh et al. 2021). However, this state is not solely driven by chronological aging (Franck et al. 2025). Instead, diverse pathophysiological stressors, including metabolic dysregulation, oxidative stress, and genomic instability, can precipitate premature immunosenescence (Rodrigues et al. 2021). A defining hallmark of immunosenescence is the accumulation of T cells characterized by irreversible cell cycle arrest and functional abnormalities (Mittelbrunn and Kroemer 2021). Rather than being merely inert, these senescent cells acquire a senescence‐associated secretory phenotype (SASP) (Wang et al. 2024), releasing pro‐inflammatory cytokines and chemokines that amplify tissue damage and perpetuate chronic mucosal inflammation (Birch and Gil 2020).

Ulcerative colitis (UC), a predominant form of inflammatory bowel disease (IBD), is characterized by unchecked colonic mucosal inflammation, with pathogenic T cells contributing substantially to tissue injury (Hracs et al. 2025; Zundler et al. 2019; Mitsialis et al. 2020). Despite the availability of various therapeutic agents, high rates of primary non‐response underscore a critical blind spot in our understanding of T cell dysfunction in UC (Peyrin‐Biroulet et al. 2024). While classical IBD therapeutic agents predominantly focus on T cell hyperactivation or recruitment (Papamichael et al. 2022), the potential involvement of premature T cell senescence has been largely overlooked. Consequently, whether T cell senescence drives UC pathogenesis or represents a viable therapeutic target in UC remains largely unexplored.

Here, we delineate premature immunosenescence as a core pathogenic vulnerability in UC, identifying a specific subset of senescent CD8^+^ T cells as the primary driver of intestinal inflammation. Mechanistically, we uncover that NAD^+^ metabolic dysregulation promotes CD8^+^ T cell senescence via a mitochondrial dysfunction‐associated cGAS‐STING signaling. Furthermore, we provide evidence that clearance of senescent CD8^+^ T cells using senolytic therapy significantly ameliorates colitis. Clinically, we find that high infiltrations of senescent CD8^+^ T cells and NAD^+^ dysregulation not only correlate with disease severity but also predict primary resistance to frontline biologic therapies. Collectively, our findings unveil the NAD^+^‐cGAS‐STING axis as a fundamental instigator of T cell senescence, offering targeted senolysis as a highly promising precision medicine paradigm for refractory UC.

## 2 Results

### 2.1 Ulcerative Colitis Exhibits Intestinal T Cells Senescence

**FIGURE 1:**
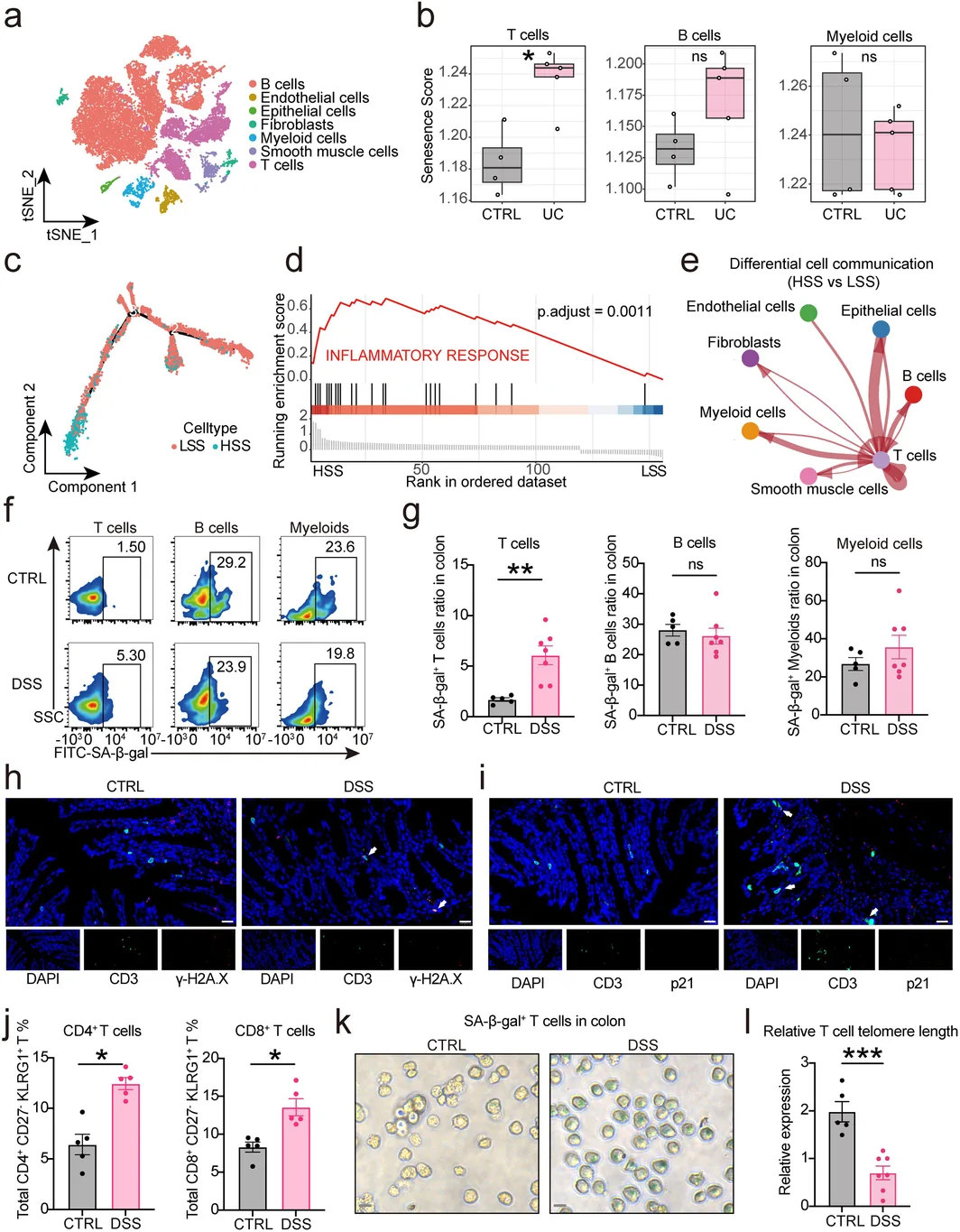
Intestinal T Cell Senescence is a Feature of Ulcerative Colitis. (a) tSNE plot of scRNA‐seq data from intestinal immune cells of UC patients and healthy controls (HC). (b) Senescence module scores of intestinal T cells, B cells, and myeloid cells in UC patients versus healthy controls. (c) Monocle pseudotime trajectory of T cells. (d) Gene Set Enrichment Analysis (GSEA) results of inflammatory response pathways in HSS versus LSS T cells. (e) Differential cell–cell communication between T cell subgroups and other intestinal cell types. (f–g) Representative flow cytometry plots and statistical analysis of SA‐β‐gal staining in intestinal immune cells. CTRL (*n* = 5); DSS (*n* = 7). (h–i) Representative immunofluorescence staining of γ‐H2A.X^+^ CD3^+^ T cells (h) and p21^+^ CD3^+^ T cells (i) in colonic tissues from CTRL and DSS‐treated mice, scale bar = 20 μm. (j) Flow cytometry analysis showing the proportion of CD27^−^ KLRG1^+^ senescent T cells in the intestines (*n* = 5). (k) SA‐β‐gal staining of intestinal T cells, scale bar = 20 μm. (l) Telomere length analysis of intestinal T cells. CTRL (*n* = 5); DSS (*n* = 7). Error bars represent SEM. ns, not significant; *p < 0.05; **p < 0.01; ***p < 0.001.

To investigate intestinal immunosenescence in UC, we analyzed scRNA‐seq data from the colonic mucosa of UC patients and healthy controls (GSE182270) (Uzzan et al. 2022). Following cell type annotation (Figure 1a), senescence scoring based on established gene signatures (de Magalhaes et al. 2024) (Table S1) revealed significantly higher senescence‐module scores in T cells from UC patients, whereas B cells and myeloid cells exhibited no such increase (Figure 1b). Crucially, these results were confirmed in an independent validation cohort (GSE214695) (Garrido‐Trigo et al. 2023), supporting the accumulation of senescent T cells within the UC microenvironment (Figure S1a,b). T cells were stratified into a high‐senescence score (HSS) group (top 10%) and a low‐senescence score (LSS) group (remaining 90%). Pseudotime analysis positioned HSS T cells at the terminal state, indicative of terminal differentiation (Figure 1c). Furthermore, Gene set enrichment analysis (GSEA) showed that the inflammatory‐response pathway was significantly enriched in HSS T cells (Figure 1d). Cell–cell communication analysis exhibited that HSS T cells maintained markedly enhanced interactions with other intestinal cells, particularly epithelial cells, suggesting a pathogenic role (Figure 1e).

To validate these findings in vivo, we utilized a chronic colitis mouse model induced by cyclical 3% DSS administration (Figure S1c). Colitis mice exhibited canonical disease phenotypes, including weight loss, colon shortening, and severe mucosal ulceration with crypt architectural distortion (Figure S1d,e). Immune cells were then isolated from the spleen, mesenteric lymph nodes (MLNs), and colonic lamina propria for flow cytometry analysis (gating strategy in Figure S1f). The results showed a stark increase in the frequency of SA‐β‐gal^+^ senescent T cells in the inflamed colon (Figure 1f,g). Furthermore, we observed an elevated proportion of SA‐β‐gal^+^ T cells in the spleens and MLNs of colitis mice, although this increase was less pronounced compared to that in the colon (Figure S1g–j). Immunofluorescence staining revealed an accumulation of both γ‐H2A.X^+^ CD3^+^ and p21^+^ CD3^+^ T cells in the colons of colitis mice compared to controls (Figure 1h–i), providing complementary evidence of DNA damage and cell cycle arrest features. In addition, the proportions of both CD27^−^ KLRG1^+^ CD4^+^ and CD27^−^ KLRG1^+^ CD8^+^ T cells were increased in colitis mice (Figure 1j). By contrast, the frequency of SA‐β‐gal^+^ B cells and myeloid cells was not significantly altered (Figure 1f,g, Figure S1g–j). Further, isolated colonic T cells from DSS‐treated mice displayed elevated intrinsic SA‐β‐gal activity (Figure 1k) and shorter telomeres (Figure 1l). Collectively, these results support an enrichment of senescence‐associated features in colonic T cells during chronic colitis.

### 2.2 Senolytic Therapy Alleviates Colitis by Clearing Senescent CD8 ^+^ T Cells

**FIGURE 2:**
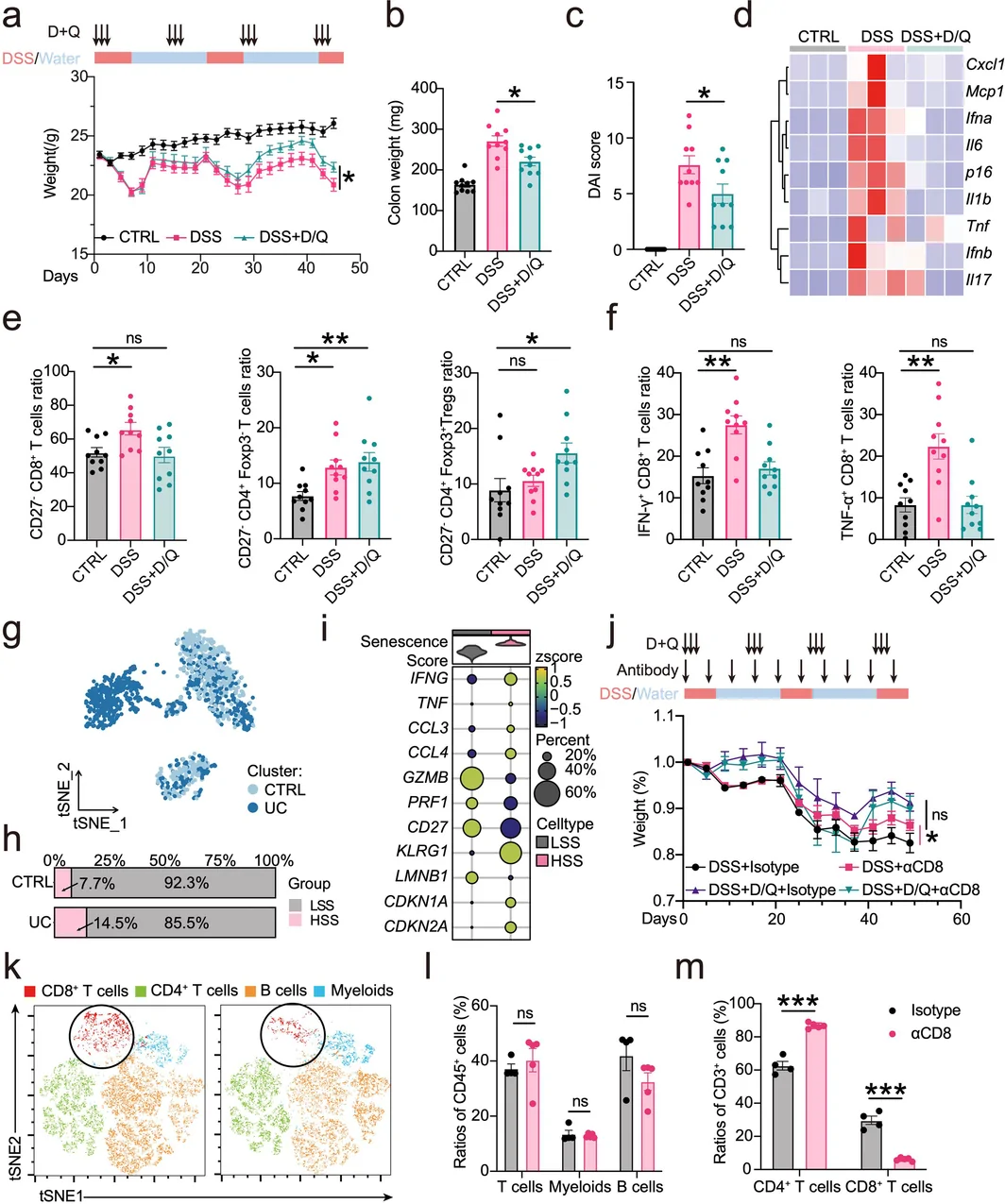
Senolytic Therapy Alleviates Colitis by Selectively Eliminating Senescent CD8^+^ T Cells. (a) Schematic of the animal model and mice body weight (*n* = 10). (b, c) Bar plot of colon weight and Disease Activity Index (DAI) scores. (d) Heatmap of inflammatory and senescence‐associated genes in colon tissue. (e, f) Flow cytometry analysis showing the ratios of CD27^−^ cells in different T cell subsets (CD8^+^ T, CD4^+^ T, CD4^+^ Foxp3^+^ T) and the ratios of IFN‐γ^+^ and TNF‐α^+^ producing CD8^+^ T cells. (g) tSNE plot of intestinal CD8^+^ T cells from patients. (h) Proportions of HSS and LSS CD8^+^ T cells. (i) Dot plot of pro‐inflammatory cytokines, cytotoxic molecules and senescence‐related genes. (j) Experimental scheme and body‐weight trajectories for D + Q treatment and CD8 depletion. (k‐m) tSNE plot and quantification of colon immune cell frequencies, Isotype (*n* = 4); αCD8 (*n* = 5). Error bars represent SEM. ns, not significant; *p < 0.05; **p < 0.01; ***p < 0.001.

To investigate the pathogenic contribution of senescent T cells to UC, we evaluated the efficacy of senolytic therapy using a well‐characterized regimen of dasatinib and quercetin (D + Q) (Novais et al. 2021). In mice with chronic colitis, D + Q administration significantly attenuated body weight loss, suppressed disease activity index (DAI), and prevented chronic colitis‐associated increases in colon weight compared to vehicle‐treated controls (Figure 2a–c). Moreover, the expression of proinflammatory cytokines, including *Tnf*, *Il6*, *Il1b*, *Mcp1*, and *Cxcl1*, was significantly downregulated (Figure 2d). Successful downregulation of the senescent burden was verified by a significant reduction in the classical senescence markers *Cdkn2a* (p16) and *Cdkn1a* (p21) (Figure S2a,b). These findings indicate that senolytic therapy effectively mitigates cellular senescence and ameliorates colitis symptoms.

Next, we analyzed immune cells isolated from the colonic lamina propria via flow cytometry. We observed a significant decrease in the frequency of CD27^−^ CD8^+^ T cells following D + Q treatment, whereas the proportions of CD27^−^ CD4^+^ T cells and CD27^−^ CD4^+^ Foxp3^+^ cells remained unaltered (Figure 2e). Underscoring their pathogenic relevance, the frequency of CD27^−^ CD8^+^ T cells, but not CD27^−^ CD4^+^ T cells, strongly correlated with disease severity metrics, including body weight and colon weight (Figure S2c,d). Functionally, D + Q treatment profoundly reduced the production of IFN‐γ and TNF‐α by colonic CD8^+^ T cells (Figure 2f). These data suggest that senescent CD8^+^ T cells constitute a key population driving intestinal inflammation severity in UC.

To molecularly characterize this pathogenic subset, we sub‐clustered CD8^+^ T cells from the scRNA‐seq dataset (GSE182270) for further analysis (Figure 2g). Colonic CD8^+^ T cells from UC patients exhibited significantly higher senescence scores compared to HC (Figure S2e). Consistently, the proportion of HSS CD8^+^ T cells was markedly expanded in the inflamed microenvironment (Figure 2h). Transcriptomic profiling revealed that HSS CD8^+^ T cells exhibit a prominent SASP. This state was characterized by upregulated pro‐inflammatory mediators (*IFNG*, *TNF*, *CCL3*, *CCL4*) and senescence associated genes (*CDKN2A*, *CDKN1A*, *KLRG1*), alongside lower expression of canonical cytotoxic molecules (*GZMB*, *PRF1*) (Figure 2i). Importantly, this senescent state was phenotypically distinct from T cell exhaustion. Classical exhaustion checkpoint markers (*PDCD1*, *CTLA4*, *LAG3*, *HAVCR2*) showed no differential expression between the HSS and LSS CD8^+^ T cells (Figure S2f). Additionally, pathway analysis revealed that chronic inflammatory responses and negative regulation of wound healing signatures were significantly enriched in HSS CD8^+^ T cells (Figure S2g). These results further support a pathogenic role for these cells in UC.

To examine the contribution of CD8^+^ T cells to the therapeutic effect associated with D + Q, we conducted an in vivo depletion experiment using D + Q, an anti‐CD8 neutralizing antibody, or a combination of both (Figure 2j–m). While all three interventional strategies successfully restored body weight and diminished DAI scores compared to vehicle‐treated colitis mice, the combination therapy yielded no additive therapeutic benefit over either single‐agent treatment (Figure 2j). These results reveal that the therapeutic amelioration of colitis by senolytics is primarily mediated by the clearance of senescent CD8^+^ T cells.

### 2.3 Ulcerative Colitis Exhibits Dysregulated Intestinal NAD ^+^ Metabolism

**FIGURE 3:**
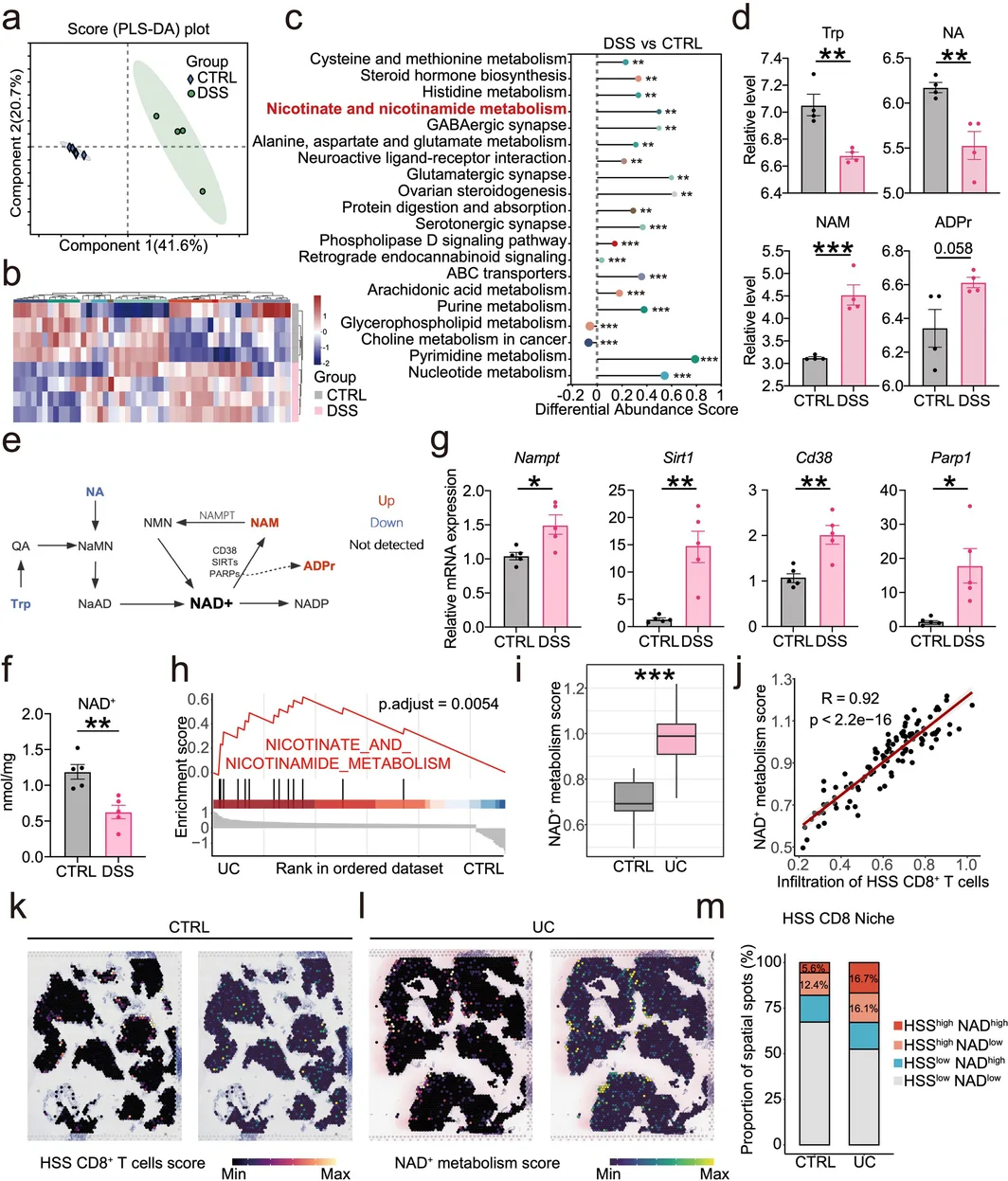
Intestinal NAD^+^ Metabolism is Significantly Dysregulated in Ulcerative Colitis. (a) PLS‐DA plot in the intestinal metabolic profile of colitis and control mice (*n* = 4). (b) Heatmap of differentially abundant colonic metabolites. (c) KEGG pathway enrichment analysis from intestinal metabolites of colitis and control mice. (d) Barplots showing NAD^+^ synthesis precursors (Trp, NA), catabolites (NAM, ADPr) (*n* = 4). (e) Schematic of the metabolic changes within the NAD^+^ metabolic pathway. (f) NAD^+^ analysis from colon of colitis and control mice. (g) Barplots showing mRNA expression of *Nampt*, *Sirt1*, *Cd38*, and *Parp1* (*n* = 5). (h) GSEA plot of enriched nicotinate and nicotinamide metabolism pathway in the intestinal metabolites of UC patients. (i) NAD^+^ metabolic pathway score of intestinal metabolites from UC patients and healthy control. (j) Correlation analysis of infiltration score of HSS CD8^+^ T cells and NAD^+^ metabolism score of public UC dataset. (k, l) Spatial transcriptomic of human colonic mucosa (GSE189184) illustrating the spatial distribution of the HSS CD8^+^ T cell signature (left columns) and the NAD^+^ metabolism signature (right columns) in representative CTRL (k) and UC (l) tissue sections. (m) Quantitative stratification of the colonic microenvironment into distinct ecological niches. Error bars represent SEM. HSS, high senescence score; ns, not significant; *p < 0.05; **p < 0.01; ***p < 0.001.

Alongside pro‐inflammatory signaling, multiple metabolism‐related pathways were upregulated in HSS CD8^+^ T cells (Figure S2g). Given that metabolic dysfunction is a recognized hallmark of cellular senescence (Herranz and Gil 2018; Hernandez‐Segura et al. 2018; Kim and Dixit 2025), we hypothesized that CD8^+^ T cell senescence in colitis might be driven by metabolic alterations. Untargeted metabolomic profiling of the murine intestinal mucosa demonstrated a distinct metabolic divergence between colitis mice and healthy hosts, as evidenced by partial least squares discriminant analysis (PLS‐DA) (Figure 3a). Pathway enrichment analysis of differentially abundant metabolites highlighted significant enrichment of the nicotinate and nicotinamide metabolism pathway in the colitis group (Figure 3b,c). This dysregulation was characterized by a reduction of NAD^+^ biosynthetic precursors (tryptophan [Trp] and nicotinic acid [NA]) and an accumulation of its catabolites (nicotinamide [NAM] and ADP‐ribose [ADPr]) (Figure 3d,e). Consistent with these metabolomic shifts, direct quantification confirmed reduced NAD^+^ levels in the inflamed colons (Figure 3f). Concurrently, the expression of key NAD^+^‐consuming and salvage enzymes (*Nampt*, *Sirt1*, *Cd38*, and *Parp1*) was significantly upregulated (Figure 3g), indicating a hyperconsumptive state driving NAD^+^ exhaustion.

To validate these findings in humans, we analyzed clinical mucosal transcriptomes (GSE87466) (Li et al. 2018). GSEA corroborated that the nicotinate and nicotinamide metabolism pathway was significantly enriched in UC patients (Figure 3h). Using single‐sample gene set enrichment analysis (ssGSEA), we found that the NAD^+^ metabolism pathway score was significantly higher in UC patients than in healthy controls (Figure 3i). Notably, the infiltration score of HSS CD8^+^ T cells positively correlated with the NAD^+^ metabolism pathway score in UC patients (Figure 3j). To map this interaction within the tissue architecture, we analyzed human colonic spatial transcriptomics data (GSE189184) (Gupta et al. 2024). By evaluating the spatial co‐localization of HSS CD8^+^ T cells and NAD^+^ metabolism dysregulation, we found that healthy colons were dominated by an HSS^low^ NAD^low^ healthy niche, with the HSS^high^ NAD^high^ senescent niche being relatively rare (5.6%). In contrast, this HSS^high^ NAD^high^ senescent niche expanded dramatically to 16.7% in UC tissues (Figure 3k–m). These findings indicated that the emergence of senescent CD8^+^ T cells was spatially associated with local mucosal niches showing high NAD^+^ metabolic scores. Importantly, previous studies have demonstrated that NAD^+^ precursors supplementation alleviated DSS‐induced colitis (Huang et al. 2022). Taken together, these findings suggest that NAD^+^ dysregulation may contribute to CD8^+^ T cells senescence in UC.

### 2.4 Reduced NAD ^+^ Availability Promotes CD8 ^+^ T Cells Senescence

**FIGURE 4:**
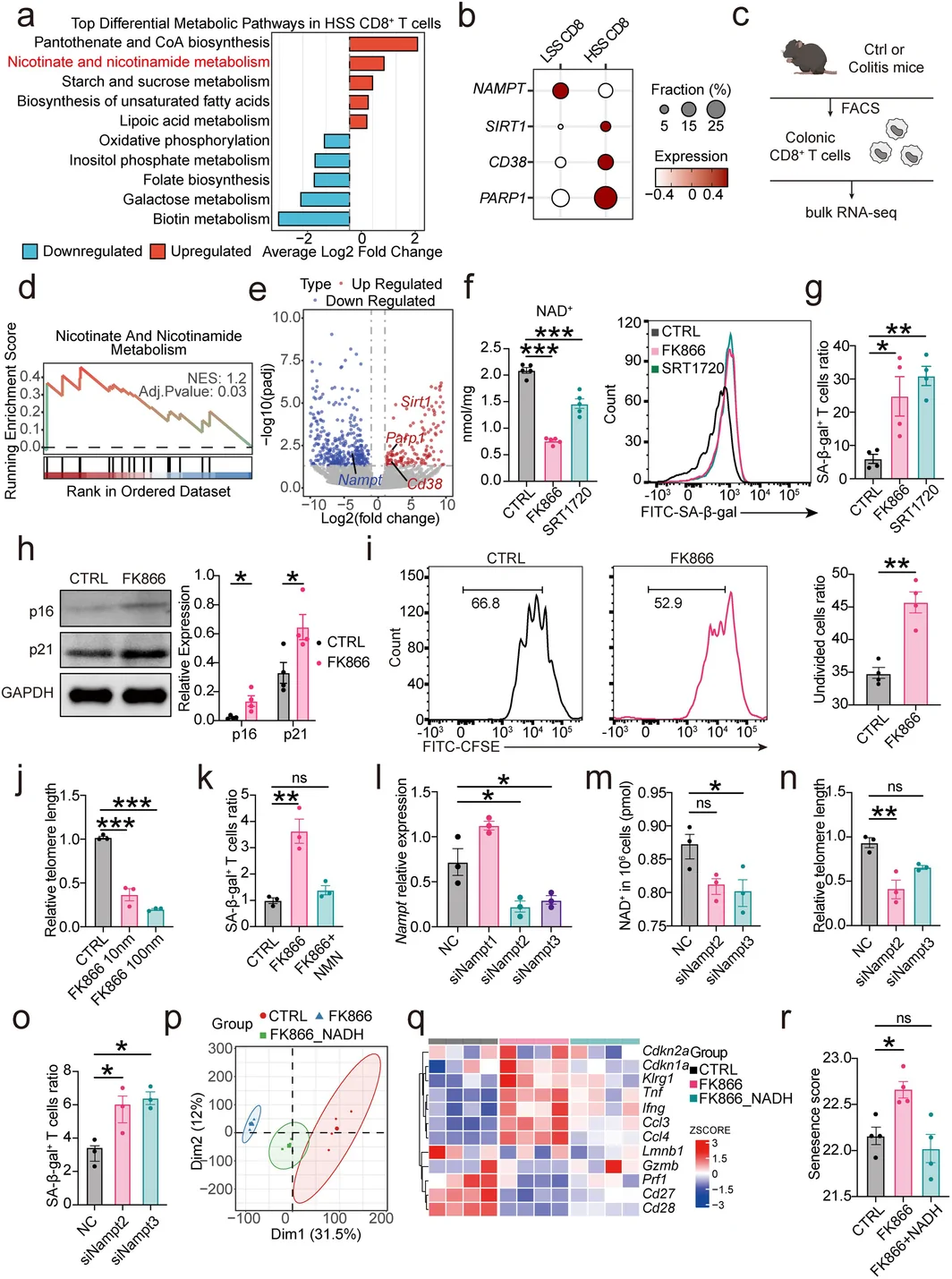
Reduced NAD^+^ Levels Induce CD8^+^ T Cell Senescence. (a) Bar plot displaying the top differentially KEGG metabolic pathways in HSS CD8^+^ T cells. (b) Dot plot showing the expression of *NAMPT*, *SIRT1*, *CD38*, and *PARP1* in LSS and HSS CD8^+^ T cells. (c) Schematic of the isolation of colonic CD8^+^ T cells from control and colitis mice by FACS for bulk RNA‐seq. (d) GSEA showing enrichment of the KEGG nicotinate and nicotinamide metabolism pathway in colonic CD8^+^ T cells from colitis mice. (e) Volcano plot of differentially expressed genes, with *Nampt*, *Sirt1*, *Parp1*, and *Cd38* highlighted. (f) Measurement of intracellular NAD^+^ levels in CD8^+^ T cells treated with vehicle, 100 nm FK866 or 10 μm SRT1720 for 72 h (*n* = 5). (g) Flow cytometry histogram and statistical analysis of SA‐β‐gal^+^ senescent CD8^+^ T cells under different treatments (*n* = 4). (h) Western blot results of p16 and p21 proteins in CD8^+^ T cells before and after FK866 treatment (*n* = 4). (i) Histogram showing the CFSE staining results of CD8^+^ T cells before and after FK866 treatment (*n* = 4). (j) Measurement of telomere length in CD8^+^ T cells after treatment with different concentrations of FK866 (*n* = 4). (k) Bar plot showing the ratio of SA‐β‐gal^+^ senescent CD8^+^ T cells under different treatments. (*n* = 3) (l–o) Validation results of NAMPT knockdown via siRNA (*n* = 3). The panels showing NAMPT mRNA expression (l), intracellular NAD^+^ levels (m), telomere length (n), and the ratio of SA‐β‐gal^+^ senescent cells (o) in CD8^+^ T cells. (p–r) Bulk RNA‐seq results of CD8^+^ T cells under different treatments (*n* = 4). PCA plot (p) showing the transcriptional profiles of the different treatment groups; Heatmap (q) illustrating the expression of senescence‐ and T cell function‐related genes; and bar plot (r) displaying the senescence scores for each group (*n* = 4). Error bars represent SEM. ns, not significant; *p < 0.05; **p < 0.01; ***p < 0.001.

To corroborate that NAD^+^ metabolic reprogramming is an intrinsic hallmark of senescent CD8^+^ T cells, we performed single‐cell metabolic pathway scoring. Differential activity analysis revealed that the nicotinate and nicotinamide metabolism pathway was profoundly upregulated in HSS CD8^+^ T cells compared to their LSS counterparts (Figure 4a). Additionally, HSS CD8^+^ T cells displayed significantly reduced expression of *NAMPT*, alongside markedly elevated levels of *CD38*, *SIRT1*, and *PARP1* (Figure 4b). To validate this intrinsic metabolic reprogramming, we performed bulk RNA‐seq on colonic CD8^+^ T cells sorted from colitis mice and healthy controls (Figure 4c). GSEA and expression profiling confirmed a significant enrichment of the NAD^+^ metabolism pathway, driven by downregulated *Nampt* and robustly upregulated *Cd38*, *Sirt1*, and *Parp1* (Figure 4d,e). Therefore, we investigated whether intracellular NAD^+^ depletion promotes CD8^+^ T cell senescence in vitro. Primary murine CD8^+^ T cells were treated with FK866 (an inhibitor of the NAD^+^ salvage enzyme NAMPT) or SRT1720 (an agonist of the NAD^+^‐consuming deacetylase SIRT1). Both pharmacological interventions effectively downregulated intracellular NAD^+^ levels and significantly expanded the SA‐β‐gal^+^ senescent population, indicating that lowering intracellular NAD^+^ is sufficient to induce CD8^+^ T cell senescence (Figure 4f,g). Given the pleiotropic cellular effects of SIRT1 (Shen et al. 2024), we focused on NAMPT inhibition via FK866 for subsequent experiments to specifically restrict NAD^+^ availability. FK866 treatment induced multiple senescence‐associated features, evidenced by the robust upregulation of cell‐cycle arrest proteins p16 and p21 (Figure 4h), severely impaired proliferative capacity (Figure 4i), and accelerated telomere attrition (Figure 4j). Subsequently, we supplemented CD8^+^ T cells with the NAD^+^ precursor NMN prior to FK866 exposure. NMN largely abrogated FK866‐induced senescence (Figure 4k). Importantly, this pharmacological phenotype was reproduced by siRNA‐mediated *Nampt* knockdown, which similarly depleted NAD^+^ pools, shortened telomeres, and upregulated SA‐β‐gal activity (Figure 4l–o).

To investigate the global transcriptomic rewiring associated with NAD^+^ reduction, we performed bulk RNA‐seq. Principal component analysis (PCA) highlighted a distinct transcriptomic divergence in FK866‐treated cells, which shifted toward the control profile after NAD^+^ supplementation (Figure 4p). Heatmap analysis revealed that FK866‐treated cells upregulated senescence‐associated cell cycle inhibitors (*Cdkn2a*, *Cdkn1a*) and the terminal differentiation marker *Klrg1*, while downregulating co‐stimulatory molecules (*Cd27*, *Cd28*) and cytotoxic molecules (*Gzmb*, *Prf1*). Conversely, inflammatory cytokines (*Tnf*, *Ifng*, *Ccl3*, *Ccl4*) were highly expressed (Figure 4q). Pathway analysis showed significant enrichment of senescence‐related pathways, such as p53 and MAPK, as well as pro‐inflammatory TNF‐α and NF‐κB signaling in the FK866‐treated group (Figure S3a). Based on ssGSEA, the FK866 group exhibited a significantly higher senescence score compared to controls (Figure 4r).

Flow cytometry functionally validated this transcriptomic shift, demonstrating enhanced IFN‐γ and TNF‐α secretion but reduced perforin production and impaired EdU incorporation (Figure S3b–f). FK866‐treated cells skewed away from an effector state (CD44^high^ CD62L^low^) toward a terminally differentiated central memory‐like phenotype (CD44^high^ CD62L^high^), without upregulating the canonical exhaustion marker PD‐1 (Figure S3g–i). We also utilized CD8^+^ T cells from OT‐1 mice to assess antigen‐dependent activation. While FK866 robustly induced SA‐β‐gal activity and impaired perforin production and proliferation in OT‐1 cells (Figure S3j–l), it surprisingly blunted their capacity to secrete IFN‐γ upon cognate antigen (OVA peptide) stimulation (Figure S3m). These results indicate that intracellular NAD^+^ depletion paralyzes antigen‐specific TCR responses, while simultaneously triggering an antigen‐independent pro‐inflammatory program. Collectively, these experiments establish NAD^+^ metabolic reduction as a direct and potent driver of CD8^+^ T cell senescence.

### 2.5 Adoptive Transfer of NAD ^+^‐Depleted Senescent CD8 ^+^ T Cells Exacerbates Colitis In Vivo

**FIGURE 5:**
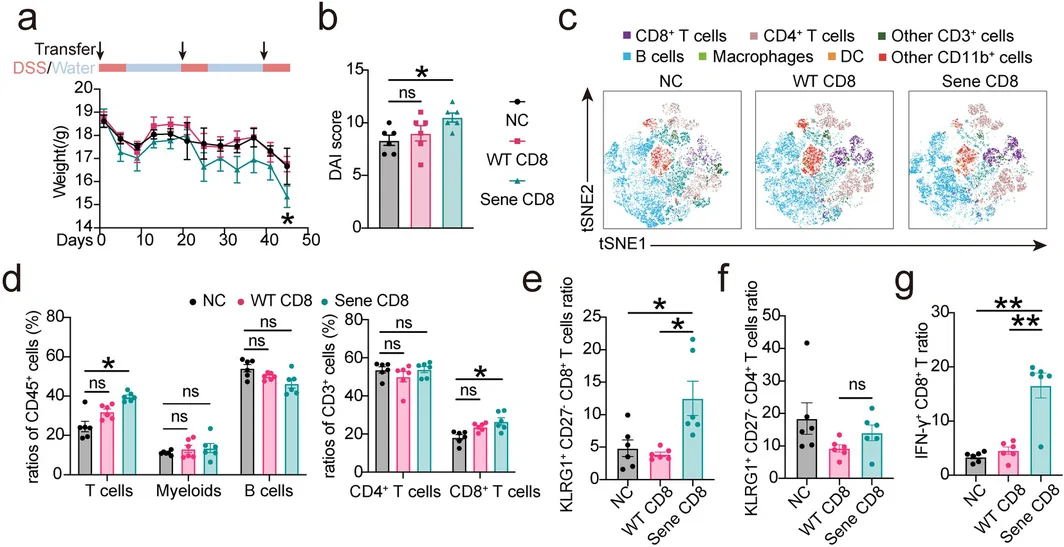
Adoptive Transfer of NAD^+^ Dysregulation‐Induced Senescent CD8^+^ T Cells Directly Exacerbated Colitis. (a, b) A schematic diagram of the adoptive transfer experiment (a) and DAI at the experimental endpoint (b). Donor CD8^+^ T cells were treated with vehicle or FK866 (100 nM, 72 h) (*n* = 6). (c, d) Flow cytometry tSNE plot of colonic immune cells (c) and statistical plot of immune cell ratios (d) (*n* = 6). (e, f) Frequencies of CD27^−^ KLRG1^+^ CD8^+^ T‐cells (e) and CD4^+^ T cells (f) in the colon (*n* = 6). (g) Frequencies of IFN‐γ^+^ CD8^+^ T cells in the mouse intestine under different treatments (*n* = 6). Error bars represent SEM. ns, not significant; *p < 0.05; **p < 0.01.

To verify the direct pathogenic role of senescent CD8^+^ T cells in vivo, we performed adoptive transfer of FK866‐induced senescent CD8^+^ T cells into mice with DSS‐induced colitis. Strikingly, mice receiving FK866‐treated CD8^+^ T cells experienced significantly accelerated disease progression, characterized by more severe weight loss and higher DAI scores when compared to both the vehicle‐transferred colitis group and those receiving control CD8^+^ T cells (Figure 5a,b). Flow cytometric analysis of colonic lamina propria cells (Figure 5c) revealed a pronounced expansion of the total T cells compartment in the senescent‐transfer recipients (Figure 5d). This expansion was mainly driven by the accumulation of CD8^+^ T cells, while the frequencies of CD4^+^ T cells, myeloid cells, and B cells remained unchanged across all groups (Figure 5d). These data confirmed the successful engraftment and retention of transferred cells in the inflamed colon. Phenotypic analysis showed that the frequency of KLRG1^+^ CD27^−^ senescent CD8^+^ T cells was significantly increased in the senescent‐transfer group (Figure 5e), whereas the frequency of KLRG1^+^ CD27^−^ CD4^+^ T cells was unaffected (Figure 5f). Furthermore, this influx of senescent CD8^+^ T cells translated into a significantly elevated frequency of IFN‐γ‐producing CD8^+^ T cells within the inflamed mucosa (Figure 5g). Together, these data demonstrate that NAD^+^ dysregulation‐driven senescent CD8^+^ T cells, characterized by an enhanced pro‐inflammatory phenotype, directly exacerbate intestinal inflammation in vivo.

### 2.6 NAD ^+^ Dysregulation Promotes CD8 ^+^ T Cell Senescence via Mitochondrial Damage‐Associated cGAS‐STING Activation

**FIGURE 6:**
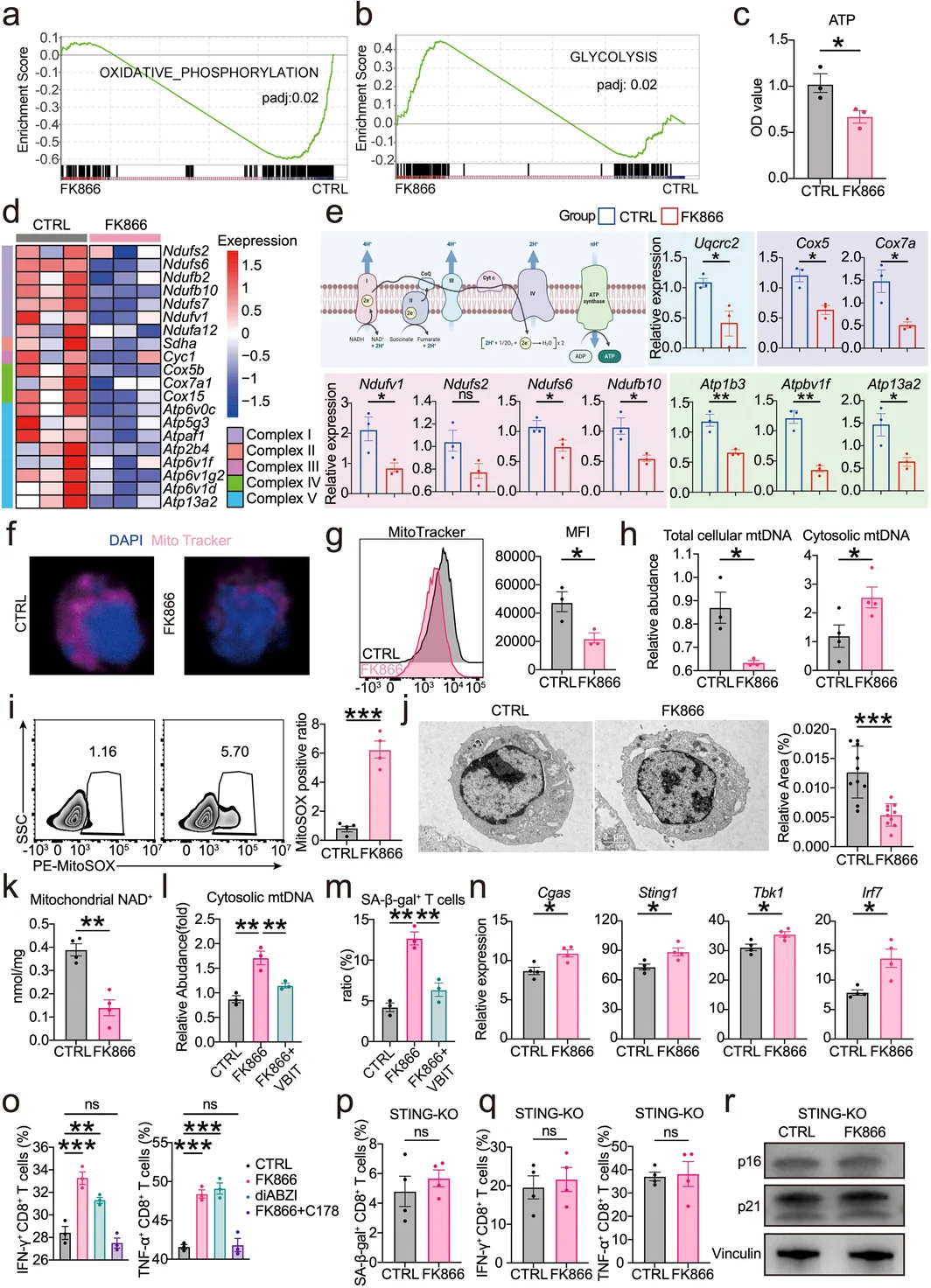
NAD^+^ dysregulation promotes T cell senescence via a mitochondrial‐cGAS‐STING Axis. (a, b) GSEA of oxidative phosphorylation (a) and glycolysis (b) in FK866‐treated versus control CD8^+^ T cells. (c) Intracellular ATP levels in CD8^+^ T cells (*n* = 3). (d, e) RNA‐seq (d) and qPCR (e) results showing the expression levels of genes related to the mitochondrial electron transport chain in CD8^+^ T cells with or without after FK866 treatment (*n* = 3). (f, g) Representative MitoTracker immunofluorescence images (f) and flow cytometry analysis (g) of CD8^+^ T cells before and after FK866 treatment (*n* = 3). (h) Abundance of total mtDNA and cytoplasmic mtDNA in CD8^+^ T cells before and after FK866 treatment (*n* = 4). (i) Representative flow cytometry plots and statistical results of MitoSOX staining of CD8^+^ T cells before and after FK866 treatment (*n* = 4). (j) Representative transmission electron microscopy images and statistical analysis of mitochondrial area in CD8^+^ T cells (*n* = 10, scale bar = 2 μm). (k) Mitochondrial NAD^+^ levels in CD8^+^ T cells (*n* = 3). (l) Relative abundance of cytosolic mtDNA in CD8^+^ T cells across different treatments (*n* = 3). (m) Ratio of SA‐β‐gal^+^ senescent CD8^+^ T cells following indicated treatments (*n* = 3). (n) RNA expression levels of key cGAS‐STING pathway genes in CD8^+^ T cells before and after FK866 treatment (*n* = 4). (o) Quantitative flow cytometry results showing the ratios of IFN‐γ^+^ and TNF‐α^+^ CD8^+^ T cells after different treatments (*n* = 3). (p‐q) Frequencies of SA‐β‐gal^+^ senescent cells (p) and IFN‐γ^+^/TNF‐α^+^ (q) STING‐KO CD8^+^ T cells after vehicle or FK866 treatment (*n* = 4). (r) Western blot analysis of p16 and p21 expression in STING‐KO CD8^+^ T cells after FK866 treatments. Error bars represent SEM. ns, not significant; *p < 0.05; **p < 0.01; ***p < 0.001.

To delineate the mechanism driving NAD^+^ depletion‐induced CD8^+^ T cell senescence, we reanalyzed the transcriptomic data of FK866‐treated cells. We found NAD^+^ depletion significantly altered the overall cellular metabolic profile (Figure S4a). GSEA revealed suppressed oxidative phosphorylation (OXPHOS) but upregulated glycolysis in FK866‐treated CD8^+^ T cells, indicating a metabolic shift (Figure 6a,b). Consistently, intracellular ATP levels were significantly reduced in senescent CD8^+^ T cells (Figure 6c). Both bulk RNA‐seq and qRT‐PCR confirmed significant downregulation of genes encoding mitochondrial electron transport chain (ETC) components, including subunits of the *Nduf*, *Cox*, and *Atp* families, as well as *Cyc1* and *Sdha* (Figure 6d,e). MitoTracker staining displayed a significant loss of mitochondrial mass, as validated by decreased mean fluorescence intensity (MFI) in FK866‐treated cells (Figure 6f,g). Furthermore, while total mitochondrial DNA (mtDNA) was reduced, cytosolic mtDNA levels were markedly increased in senescent CD8^+^ T cells (Figure 6h). MitoSOX staining indicated a significant accumulation of mitochondrial reactive oxygen species (mtROS) (Figure 6i). Transmission electron microscopy (TEM) captured aberrant mitochondrial morphology with significantly reduced mitochondrial area in FK866‐treated cells (Figure 6j). In addition, we isolated mitochondria of CD8^+^ T cells and quantified their internal NAD^+^ content. The results revealed that FK866 treatment profoundly reduced the NAD^+^ levels within the mitochondria (Figure 6k). To test whether mtDNA leakage contributes upstream to STING activation, we pre‐treated cells with VBIT‐4, an inhibitor of VDAC1 oligomerization (the macropore responsible for mtDNA release) (Callender et al. 2020; Liu et al. 2022). VBIT‐4 successfully abolished cytosolic mtDNA accumulation (Figure 6l) and reduced SA‐β‐gal^+^ fractions (Figure 6m). These results support that NAD^+^ exhaustion precipitates severe mitochondrial damage, culminating in mtROS overproduction and mtDNA leakage.

To link this organelle dysfunction to the senescent phenotypes, we profiled transcription factor (TF) activity. After intersecting with differentially expressed genes, we identified 30 differentially expressed TFs, including key interferon regulators such as *Irf3* and *Irf7* (Figure S4b,c). Given that IRF3 and IRF7 are downstream effectors of the cGAS‐STING axis, we postulated that the mislocalized cytosolic mtDNA might act as an endogenous danger signal to engage this DNA‐sensing machinery. Indeed, our transcriptomic data revealed concurrent upregulation across the cGAS‐STING cascade, including *Cgas*, *Sting1*, *Tbk1*, and *Irf7* (Figure 6n). Consistent with this, VBIT‐4 treatment not only reduced the expression of *Cgas* and *Sting1* but also markedly diminished the abundance of phosphorylated STING (p‐STING) and phosphorylated IRF3 (p‐IRF3) (Figure S4d,e). To experimentally validate this mechanism, we treated CD8^+^ T cells with the STING agonist diABZI or the inhibitor C178. Immunoblotting confirmed that FK866 activated the cGAS‐STING pathway, evidenced by upregulated cGAS and IRF3 expression and increased phosphorylation of STING. Notably, direct STING hyperactivation via diABZI recapitulated the senescence phenotype by inducing p16 and p21 expression. Conversely, blockade of STING with C178 rescued FK866‐induced upregulation of these markers (Figure S4f). Similarly, flow cytometry illustrated that diABZI promoted the production of inflammatory cytokines IFN‐γ and TNF‐α, while C178 intervention firmly attenuated the pro‐inflammatory phenotype induced by NAD^+^ restriction (Figure 6o). Finally, we utilized primary CD8^+^ T cells from STING‐KO mice. FK866 failed to significantly increase SA‐β‐gal^+^ proportions, upregulate IFN‐γ and TNF‐α secretion, or induce the expression of the cell‐cycle arrest proteins p16 and p21 in STING‐deficient cells (Figure 6p–r). Taken together, these results indicate that cytosolic leakage of mtDNA, resulting from mitochondrial dysfunction, activates the cGAS‐STING pathway to drive the senescent phenotype in CD8^+^ T cells.

### 2.7 The NAD ^+^‐CD8 ^+^ T Cells Senescence Axis Correlates With Clinical Disease Severity and Therapeutic Failure in UC

To investigate the clinical relevance of NAD^+^ metabolic dysregulation and CD8^+^ T cell senescence, we analyzed comprehensive intestinal transcriptomic datasets (GSE16879, Arijs et al. 2009 and GSE73661, Arijs et al. 2018). We stratified UC patients from the GSE16879 cohort based on their NAD^+^ metabolism activity scores and senescent CD8^+^ T cell infiltration scores.

**FIGURE 7:**
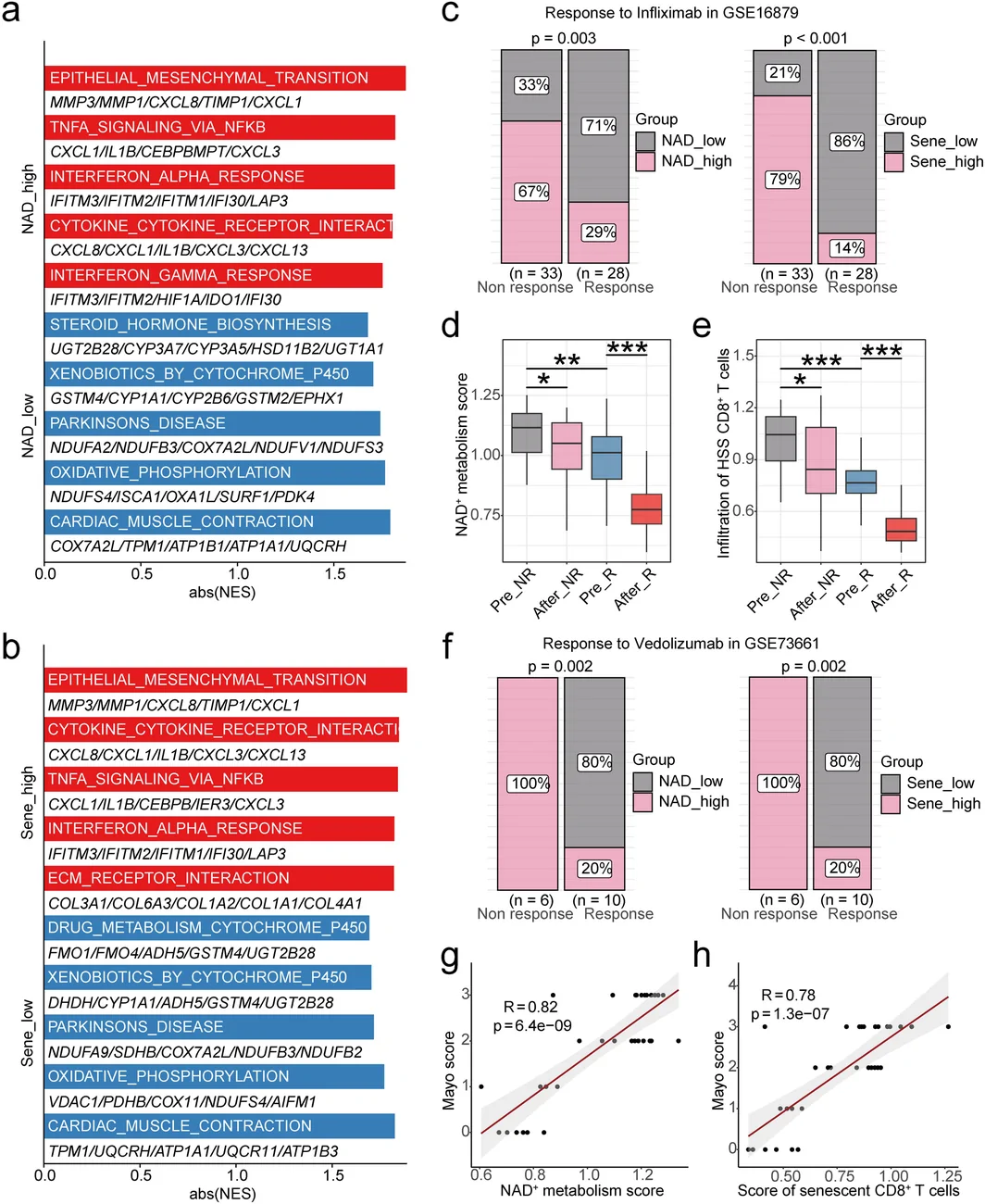
NAD^+^ Metabolic and CD8^+^ T cell Senescence Scores Predict Clinical Outcomes in Ulcerative Colitis. (a, b) GSEA results for patients stratified by high versus low NAD^+^ metabolism scores (a) or high versus low senescent CD8^+^ T‐cell infiltration scores (b). (c) Comparison of response to Infliximab treatment among different patient groups in the GSE16879 dataset. (d, e) Changes in NAD^+^ metabolic scores (d) and senescent CD8^+^ T cell infiltration scores (e) in the intestinal tissue of different UC patient groups before and after treatment. (f) Comparison of response to Vedolizumab treatment among different patient groups in the GSE73661 dataset. (g, h) Correlation plots illustrating the relationship between UC patient NAD^+^ metabolic scores (g), senescent CD8^+^ T cell infiltration scores (h), and Mayo scores. NAD_low, low NAD^+^ metabolic scores; NAD_high, high NAD^+^ metabolic scores; Sene_low, low senescent CD8^+^ T cell infiltration scores; Sene_high, high senescent CD8^+^ T cell infiltration scores; Pre_NR, Non‐Responder Pre‐treatment; After_NR, Non‐Responder After treatment; Pre_R, Responder Pre‐treatment; After_R, Responder After treatment; *p < 0.05; **p < 0.01; ***p < 0.001.

Pathway enrichment analysis revealed that patients characterized by high NAD^+^ metabolism and high senescent CD8^+^ T cells infiltration displayed significant upregulation of pro‐inflammatory pathways, including TNFA SIGNALING via NFKB and INTERFERON ALPHA RESPONSE (Figure 7a,b). Conversely, patients scoring low for both metrics maintained a more homeostatic profile, marked by preserved oxidative phosphorylation programs (Figure 7a,b). These findings suggest that this synergistic metabolic‐immune axis contributes to severe intestinal inflammation.

To determine whether this pathogenic axis compromises standard‐of‐care treatments, we evaluated therapeutic outcomes in patients receiving the anti‐TNF‐α biologic, infliximab. Patients with high scores for both NAD^+^ metabolism and senescent CD8^+^ T cells infiltration exhibited a significantly lower response rate to infliximab (Figure 7c). Furthermore, responders had significantly lower baseline NAD^+^ metabolic and senescent CD8^+^ T cells infiltration scores compared to non‐responders. Although these scores decreased in both groups post‐treatment, the reduction was markedly more pronounced in responders (Figure 7d,e). To ensure the robustness and broad applicability of this predictive biomarker, we validated our model in an independent UC cohort (GSE73661, Arijs et al. 2018) treated with a mechanistically distinct biologic. Concordantly, high pre‐treatment NAD^+^ metabolism and senescent CD8^+^ T cells infiltration scores were associated with therapeutic non‐response following administration of the α4β7 integrin antagonist, vedolizumab (Figure 7f). Finally, direct cross‐referencing with clinical parameters uncovered a strong, positive correlation between the NAD^+^‐CD8^+^ T cells senescence metrics and endoscopic disease activity indices (Figure 7g,h). Collectively, these translational insights suggest that targeting NAD^+^ metabolism and CD8^+^ T cells senescence holds promise as a novel therapeutic strategy for UC.

## 3 Discussion

Current therapeutic strategies for UC, which rely heavily on broad‐spectrum anti‐inflammatory and immunosuppressive agents, often yield incomplete remission and are associated with systemic adverse effects (Hracs et al. 2025). Consequently, identifying novel therapeutic targets based on specific pathological mechanisms remains a critical unmet need. Here, we identify immunosenescence driven by NAD^+^ metabolic reprogramming as a hallmark of UC, highlighting senescent CD8^+^ T cells as a pivotal therapeutic target.

We initially confirmed that T cell senescence characterizes intestinal inflammation in both human UC patients and murine models, identifying senescent CD8^+^ T cells as the primary pathogenic subset. While both CD4^+^ and CD8^+^ T cells exhibited senescence signatures in the inflamed mucosa, our combined multi‐omics and functional data pinpointed the CD8^+^ subset as the strongest disease‐associated compartment. Its pathogenic potential was supported by adoptive transfer experiments, where senescent CD8^+^ T cells alone were sufficient to exacerbate colitis in vivo. The absence of a significant reduction in the CD27‐negative CD4+ compartment after D + Q suggests heterogeneity across senescence‐associated immune compartments. Biologically, CD8^+^ T cells acquire an immunosenescent phenotype much faster than their CD4^+^ counterparts, likely due to intrinsic metabolic differences (Mittelbrunn and Kroemer 2021; Martínez‐Zamudio et al. 2021). Senescent CD8^+^ T cells show a greater decline in mitochondrial mass, while senescent CD4^+^ T cells maintain metabolic diversity via lipid and glucose utilization (Callender et al. 2020). We hypothesize that this metabolic vulnerability may increase the dependence of senescent CD8^+^ T cells on Src and Bcl‐2/Bcl‐xL survival pathways, which may contribute to their apparent susceptibility to D + Q. In contrast, the metabolic flexibility of CD4^+^ T cells likely enables them to utilize alternative senescent cell anti‐apoptotic pathways and may reduce sensitivity to this regimen.

A critical distinction must be drawn between T cells senescence and exhaustion, which are two distinct dysfunctional states arising from chronic antigenic stimulation and inflammation (Zhao et al. 2020; Slaets et al. 2024). While exhaustion is an adaptive, hypofunctional state characterized by the upregulation of inhibitory checkpoints (e.g., PD‐1, CTLA‐4) (Baessler and Vignali 2024), senescence is a highly bioactive, tissue‐destructive state defined by irreversible cell‐cycle arrest and a robust SASP (Mittelbrunn and Kroemer 2021). Our findings demonstrate that the CD8^+^ T cells in colitis model exhibit a senescence‐associated profile that is distinct from canonical exhaustion. While these cells exhibit functional impairment, they lack significant upregulation of canonical exhaustion markers like PD‐1, CTLA‐4, and LAG‐3, but exhibit robust senescence‐related features, including p16, p21 and KLRG1. This distinction has implications for therapeutic interpretation. While checkpoint blockade therapy has revolutionized the treatment of T cells exhaustion in cancer (Morad et al. 2021), our findings suggest that targeting T cells senescence with senolytics may be a more logical and effective approach for managing autoimmune and chronic inflammatory diseases like UC.

At the metabolic core of this senescent fate lies the severe collapse of mucosal NAD^+^ homeostasis. Previous studies have demonstrated that age‐related systemic NAD^+^ decline is driven by the accumulation of CD38^+^ immune cells, a process closely linked to SASP (Imai and Guarente 2014; Covarrubias et al. 2020; Chini et al. 2020; Chini et al. 2019; Camacho‐Pereira et al. 2016), whereas its specific role in IBD immunopathology has remained unclear. Here, our spatial and metabolomic mapping revealed a profound NAD^+^ dysregulation within the colitis microenvironment, characterized by an imbalance of impaired biosynthesis and increased expression of NAD^+^‐consuming enzymes. Crucially, we confirmed that intracellular NAD^+^ depletion was sufficient to induce CD8^+^ T cells senescence, identifying a novel metabolic etiology for T cells dysfunction in UC and establishing a new therapeutic entry point. Interestingly, the existing literature regarding NAD^+^ in colitis is context dependent, some reports suggest that inhibiting NAD^+^ synthesis limits macrophage‐driven inflammation (Gerner et al. 2018), whereas others argue that NAD^+^ precursor supplementation (e.g., nicotinamide mononucleotide) protects the intestinal epithelium (Novak et al. 2023; Cui et al. 2022; Kim et al. 2025). Our findings reconcile this complexity by positioning CD8^+^ T cells as highly sensitive victims of mucosal NAD^+^ drainage. Consequently, rather than employing systemic NAD^+^ modulation, developing strategies that specifically target NAD^+^ metabolism in senescent CD8^+^ T cells offers a much cleaner and more precise therapeutic window.

Mechanistically, we bridged the gap between metabolic starvation and the SASP by uncovering a mitochondria‐to‐cytosol danger signaling cascade. While NAD^+^ depletion is known to promote genomic instability (Chini et al. 2024), the specific mechanism linking metabolic stress to the senescent phenotype in T cells has remained elusive. Our data suggest that NAD^+^ exhaustion precipitates mitochondrial dysfunction, resulting in the leakage of mitochondrial DNA (mtDNA) into the cytosol. This mislocalized mtDNA can act as a danger signal that activates the cGAS‐STING pathway and triggers a robust antigen‐independent pro‐inflammatory program. Notably, this metabolic‐immune crosstalk appears to represent a conserved biological vulnerability. Consistent with recent studies reporting that NAD^+^ depletion drives an mtDNA‐mediated interferon response in fibroblasts and that NAD^+^ supplementation attenuates cGAS‐STING‐driven brain inflammation (Hou et al. 2021; Chini et al. 2025), our findings extend this emerging paradigm to mucosal immunity. Ultimately, our study firmly establishes the mtDNA‐cGAS‐STING axis as the indispensable pathway translating metabolic stress into immune senescence in UC.

Furthermore, we extended our findings to the clinical setting, showing that the NAD^+^‐senescence axis is a powerful determinant of biologic response. High baseline infiltration of senescent CD8^+^ T cells predicted primary non‐response to cornerstone biologics, including anti‐TNF‐α (infliximab) and anti‐integrin (vedolizumab) therapies. This positions the senescent CD8^+^ T cell signature not merely as a correlative metric of severity, but as an actionable prognostic biomarker. Integrating this signature into clinical workflows could eventually facilitate the early identification of refractory patients, guiding them toward personalized, senescence‐targeted interventions.

This study still has some limitations. First, while the DSS‐induced model mimics key features of human UC, it does not fully replicate the complex nature of human disease. Second, although we identify senescent CD8^+^ T cells as the primary pathogenic subset, the senolytic regimen (dasatinib and quercetin) is not T‐cell specific and may clear other senescent populations, such as fibroblasts and myeloid cells. Future studies utilizing lineage‐specific deletion models are needed to dissect the relative contributions of these cell types.

In conclusion, this study deepens our understanding of UC immunopathology and highlights a novel therapeutic avenue. Unlike traditional treatments that target downstream inflammation, our findings suggest that targeting the upstream contributor, pathogenic senescent CD8^+^ T cells, may effectively break the cycle of chronic inflammation. By identifying the NAD^+^‐mitochondria‐cGAS‐STING axis as a critical vulnerability, this work offers a highly promising precision medicine strategy to overcome therapeutic resistance and improve clinical outcomes in UC.

## 4 Methods

### 4.1 Animals

C57BL/6J mice were procured from Shanghai Jihui Laboratory Animal Care Co. Ltd. *Sting1*‐KO (Strain no. T012747) mice were purchased from GemPharmatech. Mice were maintained under specific pathogen‐free (SPF) conditions at the Department of Laboratory Animal Science, Fudan University (23°C, 50% humidity, 12‐h light/dark cycle). All animal procedures and experimental protocols were approved by the Institutional Animal Care and Use Committee (IACUC) of Fudan University (no. 2023‐HSYY‐343JZS).

### 4.2 Chronic Colitis Mouse Model and Interventions

To induce a chronic colitis model, mice were provided with 3% dextran sulfate sodium (DSS, MP biomedical) dissolved in drinking water for 7 consecutive days, followed by 14 days of distilled drinking water. This cycle was repeated twice, after which the mice received another 7 days of 3% DSS water. Mouse weight was recorded daily throughout the experiment. For senolytic intervention, mice were orally gavaged with a cocktail of 5 mg/kg dasatinib (Selleck) and 50 mg/kg quercetin (Merck) suspended in 0.5% carboxymethylcellulose sodium. The regimen was administered for 3 consecutive days starting from day 0 of the first DSS cycle, and repeated every 14 days until the experimental endpoint. For in vivo CD8^+^ T cell depletion, mice received intraperitoneal injections of 200 μg anti‐mouse CD8 neutralizing antibody (BioXCell) on day 0, with maintenance injections administered every 5 days. At the experimental endpoint, mice were euthanized under anesthesia, and relevant tissues were collected for subsequent processing.

### 4.3 Single‐Cell RNA‐Seq and Metabolic Pathway Inferring

Single‐cell RNA‐seq data from the intestinal tissue of UC patients and healthy controls (GSE182270, Uzzan et al. 2022 and GSE214695, Garrido‐Trigo et al. 2023) were downloaded from the GEO database. Data were processed using the Seurat (Hao et al. 2024) R package for quality control, normalization, and t‐distributed stochastic neighbor embedding (tSNE) for dimensionality reduction. Cell types were annotated based on established canonical markers. A senescence gene set was downloaded from the human aging genomic resources database (https://genomics.senescence.info/), and a senescence score for each immune cell was calculated using the AddModuleScore function in Seurat. To establish the specific signature for senescent CD8^+^ T cells, colonic CD8^+^ T cells were stratified into HSS (top 10%) and LSS (remaining 90%) subsets. Differential expression analysis was performed using the FindMarkers function. Genes meeting the thresholds of adjusted *p* < 0.05 and log2(fold change) ≥ 1 were classified as significantly upregulated (Table S2). This panel of upregulated genes was defined as the “HSS CD8^+^ T cell signature” and utilized for downstream analysis. T cells were arranged along a pseudotime trajectory using the Monocle (Cao et al. 2019) R package, with visualization performed using uniform manifold approximation and projection (UMAP). Cell–cell communication was assessed with the CellChat (Jin et al. 2025) R package. Furthermore, to deduce single‐cell metabolic heterogeneities, we applied the scMetabolism (Wu et al. 2022) R package.

### 4.4 Spatial Transcriptomics and Niche Stratification

To map the anatomical co‐localization of metabolic and immune signatures, human colonic spatial transcriptomics (ST) datasets (GSE189184, Gupta et al. 2024) were processed using the Seurat spatial framework. NAD^+^ metabolism signature was retrieved from the KEGG database. Spatial module scores for the HSS CD8^+^ T cells signature and the NAD^+^ metabolism signature were calculated for each spatial spot. To define local ecological microenvironments, spatial spots were stratified into distinct niches using the 75th percentile expression score of the entire cohort as a cutoff. Spots exhibiting scores ≥ the 75th percentile for both signatures were designated as the “Double High (Senescent Niche)”, whereas those scoring below the threshold for both were classified as the “Double Low (Homeostasis Niche)”.

### 4.5 Bulk Transcriptomic and Clinical Cohort Analysis

For bulk RNA‐seq, total RNA was extracted from colon tissue or pre‐treated primary T cells and quantified using a Qubit 4 Fluorometer. Libraries were generated using the Illumina Stranded mRNA Prep, Ligation kit, and sequencing was performed on an Illumina NovaSeq X Plus platform. After quality control and read mapping, read counts were generated and normalized using the FPKM method to create heatmaps. Differential gene expression analysis was conducted using the DESeq2 (Love et al. 2014) package (v1.44.0), with significance defined as an adjusted *p* < 0.05 and a |log2(fold change)| > 1.5. Gene Set Enrichment Analysis (GSEA) was performed using GSEA (Subramanian et al. 2005) software (v4.3.3) to determine the normalized enrichment score (NES) and *p*‐value for the Hallmark and Kyoto Encyclopedia of Genes and Genomes (KEGG) gene set collections. Other public transcriptomic datasets of UC patients and healthy controls, including GSE87466 (Li et al. 2018), GSE16879 (Arijs et al. 2009) and GSE73661 (Arijs et al. 2018), were downloaded from the GEO database. As all human data were obtained from public databases and fully anonymized, ethical approval from our institutional review board was exempted.

### 4.6 Untargeted Metabolomics Analysis

Untargeted high‐throughput metabolomics analysis was conducted by Shanghai Majorbio Biomedical Technology Co. Ltd. Briefly, samples stored at −80°C were weighed and homogenized in a water/chloroform/methanol solution containing a norleucine internal standard. After centrifugation, the supernatant was transferred, and the pellet was re‐extracted with methanol. The combined supernatants were evaporated under a nitrogen stream. The residue was reconstituted with omeprazole chloride solution, vortexed, and then derivatized with trifluoroacetamide before being subjected to mass spectrometry analysis.

### 4.7 In Vitro Induction of CD8 ^+^ T Cells Senescence

For in vitro induction of senescence, primary murine CD8^+^ T cells were isolated and cultured in complete RPMI‐1640 medium. To induce NAD^+^ depletion, the cells were treated with FK866 (Selleck) at a final working concentration of 100 nM for 72 h. Following the induction period, the cells were harvested for subsequent transcriptomic, metabolic, and functional assays or washed thoroughly with PBS prior to the adoptive transfer experiments.

### 4.8 NAD ^+^ Measurement

Intracellular and mitochondrial NAD^+^ levels were quantified using the Enhanced NAD^+^/NADH Assay Kit (Beyotime) according to the manufacturer's protocol. Prior to analysis, all samples were deproteinized to eliminate enzymatic interference. Absorbance was measured at 450 nm, and absolute NAD^+^ concentrations were calculated against a concurrently generated standard curve. The final values were normalized to total protein concentrations and expressed as nmol/mg protein.

### 4.9 Mitochondrial Isolation

Mitochondrial fractions were isolated from 2 × 10 (Birch and Gil 2020) primary CD8^+^ T cells using the Mitochondria Isolation Kit for Cultured Cells (Thermo Fisher) according to the manufacturer's instructions. After a final wash step, the isolated intact mitochondria were immediately processed for downstream analyses.

### 4.10 Flow Cytometry and Cell Sorting

Following digestion into a single‐cell suspension, cells from mouse tissues were incubated with anti‐mouse CD16/32 (Biolegend) for 15 min for Fc‐receptor blocking. Cells were then stained for surface markers according to the manufacturer's protocol. For intracellular staining, cells were fixed and permeabilized using a FOXP3/Transcription Factor Staining Buffer Set (eBioscience) after surface staining, and then incubated with the antibodies for intracellular or nuclear staining. For cellular senescence analysis, cells were stained with CellEvent Senescence Green (Thermo Fisher) according to the manufacturer's protocol. Samples were analyzed on a CytoFLEX (Beckman Coulter) flow cytometer, and data were processed using FlowJo software, with tSNE dimensionality reduction performed using default parameters. For cell sorting, surface‐stained cells were sorted using a MA900 Multi‐Application Cell Sorter (Sony Biotechnology) with a 70 mm chip at 4°C, ensuring a purity of > 95%.

### 4.11 Western Blotting

Cell lysates were prepared by washing cells twice in cold PBS and then lysing them in RIPA buffer supplemented with a protease inhibitor cocktail (Thermo Fisher). The lysates were centrifuged at 12,000 *g* for 15 min at 4°C. Protein concentration was determined using the bicinchoninic acid (BCA) assay (Beyotime). Proteins were separated by SDS‐PAGE on FuturePAGE 4%–20% gels (ACE Biotechnology), transferred to 0.2 μm PVDF membranes (Beyotime), and blocked with Protein Free Rapid Blocking Buffer (Epizyme) for 20 min at room temperature. Membranes were incubated with diluted primary antibodies overnight at 4°C. After three washes with Phosphate Buffered Saline with Tween 20 (PBST, Sangon), membranes were incubated with appropriately diluted secondary antibodies for 1 h at room temperature and visualized using an ImageQuan LAS 4000 mini (Cytiva).

### 4.12 Cytosolic mtDNA Quantification

Cytosolic mtDNA leakage was quantified as previously described (Bryant et al. 2022). Briefly, cells were divided into two aliquots for whole‐cell and cytosolic extraction. The cytosolic fraction was isolated by selectively permeabilizing the plasma membrane with 15 μg/mL digitonin for 10 min at 4°C, followed by centrifugation at 1000 × *g* (5 min) to remove nuclei and 21,000 × g (10 min) to remove intact mitochondria. DNA was extracted from both the whole‐cell and cytosolic fractions using DNeasy Kit (Qiagen). Cytosolic mtDNA was quantified by qPCR targeting mt‐CYTB and mt‐RNR2 and normalized to total whole‐cell DNA.

### 4.13 Quantitative Real‐Time PCR

Total RNA was extracted from treated cells using the RNAiso kit (Takara). cDNA was synthesized using the PrimeScript RT Master Mix (Takara). Quantitative real‐time PCR (qRT‐PCR) analysis was performed using ChamQ Universal SYBR qPCR Master Mix (Vazyme) on a QuantStudio 5 qPCR system (Thermo Fisher). Gene expression levels were normalized to Gapdh or Actb and determined by the ΔΔCt method.

### 4.14 Immunofluorescence Staining

For mitochondrial profiling, cells were incubated with MitoTracker (Thermo Fisher) for 30 min followed by fixation, or incubated with freshly prepared MitoSOX (Thermo Fisher) at 37°C for 30 min. For tissue immunofluorescence, OCT‐embedded colonic cryosections were sequentially fixed in 4% PFA, permeabilized with 0.2% Triton X‐100, and blocked in 5% BSA prior to incubation with the indicated antibodies. Image acquisition was performed using an Olympus FV3000 confocal microscope.

### 4.15 Transmission Electron Microscopy

Pre‐treated cells were first fixed in glutaraldehyde solution on coverslips for 12 h. The cells were then detached, processed according to a standard TEM protocol, and sectioned into 70‐nm ultrathin slices. These sections were subsequently imaged using a 120 kV transmission electron microscope.

### 4.16 Quantitative and Statistical Analysis

All statistical analyses were performed using GraphPad Prism 9. Statistical comparisons between two experimental groups were conducted using the two‐tailed Mann–Whitney test for unpaired data. For multi‐group analyses, the one‐way ANOVA was applied, supplemented by Sidak's post hoc tests. Longitudinal disease trajectories (e.g., body weight curves) were analyzed via two‐way ANOVA mixed‐effects models. Significance thresholds were defined as follows: *p* < 0.05 (*), *p* < 0.01 (**) and *p* < 0.001 (***).

## Author Contributions

M.Y., Q.Z. and M.K. conceptualized the experiments. M.Y., Q.Z., S.Z. and J.Y. performed experiments. M.Y., M.K., L.C. and L.L. analyzed the omics data. J.Z., J.L. and F.L. designed this project. J.Z., J.L. and F.L. wrote the paper. M.Y., Q.Z., and M.K. revised the paper. All authors provided intellectual contribution to this work.

## Funding

This work was supported by the National Key Research and Development Program of China (No. 2025YFA0923301 to F.L.).

## Conflicts of Interest

The authors declare no conflicts of interest.

## Supporting information


**Figure S1:** Validation of chronic colitis mouse model and systemic immunosenescence analysis. (a) tSNE plot of scRNA‐seq data (GSE214695) from intestinal immune cells of UC patients and healthy controls (HC). (b) Senescence module scores of intestinal T cells, B cells, and myeloid cells in UC patients versus healthy controls. (c) Schematic of the experimental procedure for the DSS‐induced chronic colitis mouse model. (d) Representative image and statistical analysis of colon length in colitis versus control mice. (e) Representative H&E staining of intestinal tissue. (f) Gating strategy of flow cytometry analysis. (g–j) Representative flow cytometry plots and statistical analysis of SA‐β‐gal staining in immune cells from the spleen and mesenteric lymph nodes (MLN). Error bars represent SEM. CTRL (*n* = 5); DSS (*n* = 7). ns, not significant; *p < 0.05; **p < 0.01; ***p < 0.001.

**Figure S2:** CD8^+^ T cells in Colitis Exhibit a Senescent Phenotype. (a) Immunohistochemistry showing intestinal p16 of mice. (b) mRNA expression of intestinal p16 and p21 (*n* = 4). (c, d) Correlation analysis between the ratio of CD27^−^ T cells and mouse body weight, colon weight. (e) Senescence scores of CD8^+^ T cells from control and UC samples. (f) Violin plot of exhaustion markers T cells. (g) Enrichment pathways in HSS CD8^+^ T cells. Error bars represent SEM. LSS, low senescence score; HSS, high senescence score; ns, not significant; *p < 0.05; **p < 0.01; ***p < 0.001.

**Figure S3:** Molecular and Functional Analysis of NAD^+^‐Induced T Cell Senescence. (a) KEGG pathway enrichment analysis of differentially expressed genes between the FK866‐treated and control groups. (b–m) Flow cytometry statistical results of CD8^+^ T cells before and after FK866 treatment (*n* = 3). The panels showing the ratio of IFN‐γ^+^ (b), TNF‐α^+^ (c), and Perforin^+^ (d) CD8^+^ T cells; the viability of tumor cells co‐cultured with CD8^+^ T cells (e); the ratio of EdU^+^ CD8^+^ T cells (f); the ratio of CD44^high^ CD62L^low^ (g) and CD44^high^ CD62L^high^ (h) CD8^+^ T cells; the ratio of PD1^+^ CD8^+^ T cells (i); the ratio of SA‐β‐gal^+^ CD8^+^ T cells (j); and in CD8^+^ T cells from OT‐1 mice, the ratio of EdU^+^ (k), Perforin^+^ (l), and IFN‐γ^+^ (m) cells. Error bars represent SEM. ns, not significant; *p < 0.05; **p < 0.01; ***p < 0.001.

**Figure S4:** cGAS‐STING Signaling Mediates NAD^+^ Dysregulation‐Induced CD8^+^ T Cell Senescence. (a) Heatmap showing changes in KEGG cellular metabolic pathways in CD8^+^ T cells. (b) Venn diagram illustrating the overlap between differentially expressed genes and predicted transcription factors in senescent CD8^+^ T cells. (c) Heatmap showing the expression profiles of intersected transcription factors in CD8^+^ T cells. (d) Relative mRNA expression levels of *Cgas* and *Sting1* in CD8^+^ T cells following indicated treatments (*n* = 3). (e) Western blot analysis of p‐IRF3, p‐STING, IRF3 and STING following different treatments. (f) Western blot showing the expression of senescence‐related protein and key proteins in cGAS‐STING pathway.

**Data S1:** Supplementary Tables.

## Data Availability

All RNA‐seq data of this study have been deposited in figshare at https://doi.org/10.6084/m9.figshare.31841095. Raw scRNA‐seq data of human samples from previously published studies can be accessed in the GEO database (GSE182270 (Uzzan et al. 2022) and GSE214695, Garrido‐Trigo et al. 2023). Raw spatial transcriptomic data of human samples from a previously published study are publicly available in the GEO database (GSE189184, Gupta et al. 2024). Raw bulk RNA‐seq data of human samples from previously published studies can be accessed in the GEO database (GSE87466, Li et al. 2018, GSE16879, Arijs et al. 2009 and GSE73661, Arijs et al. 2018). Any information required to reanalyze the data reported in this study is available upon request.
